# Quantitative Dynamic ^18^F-FDG PET/CT in Survival Prediction of Metastatic Melanoma under PD-1 Inhibitors

**DOI:** 10.3390/cancers13051019

**Published:** 2021-03-01

**Authors:** Christos Sachpekidis, Jessica C. Hassel, Annette Kopp-Schneider, Uwe Haberkorn, Antonia Dimitrakopoulou-Strauss

**Affiliations:** 1 Clinical Cooperation Unit Nuclear Medicine, German Cancer Research Center, 69120 Heidelberg, Germany; uwe.haberkorn@med.uni-heidelberg.de (U.H.); a.dimitrakopoulou-strauss@dkfz-heidelberg.de (A.D.-S.); 2 Department of Dermatology and National Center for Tumor Diseases, University Hospital Heidelberg, 69120 Heidelberg, Germany; Jessica.Hassel@med.uni-heidelberg.de; 3 Department of Biostatistics, German Cancer Research Center (DKFZ), 69120 Heidelberg, Germany; kopp@dkfz-heidelberg.de; 4 Department of Nuclear Medicine, University of Heidelberg, 69120 Heidelberg, Germany

**Keywords:** metastatic melanoma, PD-1 inhibitors, ^18^F-FDG, dynamic PET/CT, SUV, pharmacokinetics, compartment modeling, fractal analysis

## Abstract

**Simple Summary:**

The reliable and early during-the-course-of-treatment assessment of tumor response to the novel immunotherapeutic agents is a matter of debate, posing relevant challenges to conventional imaging modalities. In this prospective study, including 25 metastatic melanoma patients, we explored the prognostic significance of quantitative, dynamic ^18^F-fluorodeoxyglucose (^18^F-FDG) positron emission tomography/computed tomography (PET/CT) performed early during programmed cell death protein 1 (PD-1) blockade. At a median follow-up of 24.2 months, several semiquantitative and quantitative PET/CT parameters derived from tumor lesions and reference tissues had an impact on progression-free survival (PFS). In particular, ^18^F-FDG standardized uptake value (SUV_mean_, SUV_max_) and fractal dimension (FD) of melanoma lesions adversely affected PFS, while FD of the thyroid, as well as SUV_max_ and k_3_ of the bone marrow, positively affected PFS. These findings underline the potential predictive role of quantitative, dynamic, interim PET/CT—performed in combination with conventional, static, whole-body PET/CT—in metastatic melanoma patients under PD-1 blockade.

**Abstract:**

The advent of novel immune checkpoint inhibitors has led to unprecedented survival rates in advanced melanoma. At the same time, it has raised relevant challenges in the interpretation of treatment response by conventional imaging approaches. In the present prospective study, we explored the predictive role of quantitative, dynamic ^18^F-fluorodeoxyglucose (^18^F-FDG) positron emission tomography/computed tomography (PET/CT) performed early during immunotherapy in metastatic melanoma patients receiving treatment with programmed cell death protein 1 (PD-1) inhibitors. Twenty-five patients under PD-1 blockade underwent dynamic and static ^18^F-FDG PET/CT before the start of treatment (baseline PET/CT) and after the initial two cycles of therapy (interim PET/CT). The impact of semiquantitatively (standardized uptake value, SUV) and quantitatively (based on compartment modeling and fractal analysis) derived PET/CT parameters, both from melanoma lesions and different reference tissues, on progression-free survival (PFS) was analyzed. At a median follow-up of 24.2 months, survival analysis revealed that the interim PET/CT parameters SUV_mean_, SUV_max_ and fractal dimension (FD) of the hottest melanoma lesions adversely affected PFS, while the parameters FD of the thyroid, as well as SUV_max_ and k_3_ of the bone marrow positively affected PFS. The herein presented findings highlight the potential predictive role of quantitative, dynamic, interim PET/CT in metastatic melanoma under PD-1 blockade. Therefore, dynamic PET/CT could be performed in selected oncological cases in combination with static, whole-body PET/CT in order to enhance the diagnostic certainty offered by conventional imaging and yield additional information regarding specific molecular and pathophysiological mechanisms involved in tumor biology and response to treatment.

## 1. Introduction

Over the last few years, the development and introduction in the clinic of immune checkpoint inhibitors (ICIs) have radically changed the landscape in the management of metastatic melanoma. In particular, systemic treatment with the monoclonal antibodies ipilimumab, which targets the T-lymphocyte-associated protein 4 (CTLA-4), as well as nivolumab and pembrolizumab, which target the programmed cell death protein 1 (PD-1), has led to unprecedented response and survival rates in advanced melanoma and is nowadays considered the first-line therapy for unresectable stage III and IV melanoma patients [1,2,3,4,5,6,7].

At the same time, the widespread usage of these powerful therapeutic tools has raised some challenges with regard to the reliable assessment of response to them. Being a completely novel treatment, immunotherapy with ICIs is associated with responses, which can be different from conventional cytotoxic approaches, notably by generating inflammations rather than direct lysis [8]. A distinct feature of immunotherapy, compared to chemotherapy, is the more delayed response to agent administration with durable responses that may persist even after treatment cessation [9]. Other novel patterns associated with ICIs include the phenomenon of pseudoprogression, defined as an initial increase in tumor burden followed by tumor regression, the hyperprogressive disease, which is an aggressive pattern of rapid, marked disease progression, as well as dissociated responses, characterized by the regression of some lesions and the concurrent growth of other lesions or the appearance of new ones [10,11,12].

To make things more complicated, immunotherapeutic agents are also linked with the development of a “new class” of side effects that resemble autoimmune responses, the immune-related adverse events (irEs), whose spectrum is wide, affecting almost every organ of the body and can occur at any point in the treatment course [13]. These adverse events can also pose relevant interpretation challenges to standard imaging methods.

Positron emission tomography/computed tomography (PET/CT) represents a hybrid imaging modality, which combines the molecular-metabolic detail of PET—mostly utilizing the glucose analog ^18^F-fluorodeoxyglucose (^18^F-FDG) as a radiotracer, with the anatomic precision of CT. ^18^F-FDG PET/CT is the elective imaging technique in detecting metastatic disease in advanced melanoma [14,15,16,17]. It is, moreover, considered a relatively reliable tool for the monitoring of immunotherapy, although certain limitations are encountered with concern to response assessment [18,19,20,21,22,23].

In clinical routine, the evaluation of response to immunotherapy by means of PET/CT is primarily visual and subjective in nature, with quantitative—thus more objective—assessments being mainly restricted in the calculation of the semiquantitative parameter, standardized uptake value (SUV). SUV represents tissue activity within a volume of interest (VOI) corrected for injected activity and body weight, and is calculated when the tracer has reached equilibrium, usually at 60 min post-injection (p.i.). However, the generally accepted method for accurate analysis of ^18^F-FDG metabolism and pharmacokinetics is a two-tissue compartment model [24]. A prerequisite for this is the performance of full dynamic PET (dPET) studies for at least 60 min.

Data on the application of dynamic PET/CT in immunotherapy monitoring are limited [25]. The aim of the present prospective study is to investigate the predictive role of quantitative, dynamic ^18^F-FDG PET/CT performed early during immunotherapy in metastatic melanoma patients undergoing treatment with PD-1 inhibitors.

## 2. Materials and Methods

### 2.1. Patients

A total of 25 patients (12 males, 13 females; mean age 54.7 years) with unresectable, metastatic melanoma undergoing treatment with PD-1 inhibitors were enrolled in the study. PD-1 inhibitors were administered either as monotherapy (pembrolizumab, 8 patients; nivolumab, 4 patients) or as a combination treatment with CTLA-4 inhibitors (nivolumab/ipilimumab, 13 patients) (Table 1). Pembrolizumab was administered intravenously at a dose of 2 mg/kg every 3 weeks, and nivolumab was administered intravenously at a dose of 3 mg/kg every 2 weeks. The combination ICI therapy was administered as induction of 4 cycles of nivolumab (1 mg/kg) and ipilimumab (3 mg/kg) every 3 weeks, followed by single-agent nivolumab administration (3 mg/kg) every 2 weeks. The patients of this cohort have already been evaluated in another work of our group, but with a different concept, approach and analysis than in the here presented study [26]. Patients gave written informed consent to participate in the study and to have their medical records released. The study was approved by the Ethical Committee of the University of Heidelberg (S-107/2012) and the Federal Agency for Radiation Protection (Bundesamt für Strahlenschutz, Z 5–22463/2–2012-016).

### 2.2. PET/CT Data Acquisition

^18^F-FDG PET/CT was performed before the start of treatment (baseline PET/CT) and after the initial two cycles of ICIs’ administration (interim PET/CT). Data acquisition consisted of the dynamic part (dPET/CT) and the static part (whole-body PET/CT). In particular, dPET/CT studies were performed over the thorax and upper abdomen after intravenous administration of a maximum of 250 MBq ^18^F-FDG for 60 min using a 24-frame protocol (10 frames of 30 s, 5 frames of 60 s, 5 frames of 120 s and 4 frames of 600 s). Whole-body, static imaging from the head to the feet was performed in all patients with an image duration of 2 min per bed position for the emission scans after the end of the dynamic acquisition. A dedicated PET/CT system (Biograph mCT, S128, Siemens Co., Erlangen, Germany) with an axial field of view of 21.6 cm with TruePoint and TrueV, operated in a three-dimensional mode was used. A low-dose attenuation CT (120 kV, 30 mA) was used for attenuation correction of the dynamic emission PET data and for image fusion. All PET images were attenuation-corrected, and an image matrix of 400 × 400 pixels was used for iterative image reconstruction. Iterative image reconstruction was based on the ordered subset expectation maximization (OSEM) algorithm with two iterations and 21 subsets, as well as the time of flight (TOF).

### 2.3. PET/CT Data Analysis

Data analysis consisted of visual (qualitative) assessment, semiquantitative evaluation based on SUV calculations, and quantitative analysis of the dynamic ^18^F-FDG PET/CT data.

Visual analysis was based on the identification of focal sites of non-physiological ^18^F-FDG uptake greater than the background activity, which was considered consistent with melanoma lesions. All foci of increased ^18^F-FDG uptake were compared with the fused low-dose CT findings to ensure higher diagnostic accuracy of metastatic lesions. Moreover, patterns of ^18^F-FDG uptake suggestive of radiologic manifestations of irEs to immunotherapy were recognized and discerned from tumor manifestations [26].

The semiquantitative evaluation was based on volumes of interest (VOIs) and on the subsequent calculation of SUV_mean_ and SUV_max_. VOIs were drawn using the pseudo-snake algorithm of the Pmod software [27] and were placed over melanoma lesions as well as over references tissues without pathologic findings, including the thyroid gland, spleen, and bone marrow (lower thoracic spine). In patients with a very high number of ^18^F-FDG avid metastatic lesions, calculations were limited to the five most visually prominent lesions. SUV was calculated according to the formula: SUV = tissue concentration (Bq/g)/(injected dose (Bq)/body weight (g)). Moreover,% interval SUV changes as a response to treatment were calculated using the formula: SUV change (%) = (interim SUV − baseline SUV)/baseline SUV) × 100%.

Quantitative evaluation of the dynamic ^18^F-FDG PET/CT data derived from the thorax and upper abdomen was based on irregular VOIs, also drawn with a 50% isocontour mode (pseudo-snake), placed over the melanoma lesions as well as the above-mentioned reference organs (thyroid, spleen, bone marrow). The analysis was performed using a dedicated software package [28,29] and was based on a two-tissue compartment model, with methods already reported in the literature and performed previously by our group [25,30,31,32]. The two-tissue compartment model describes tracer kinetics in the studied area and involves the plasma compartment, the free (unbound) component of ^18^F-FDG in interstitial space, and the phosphorylated component of the radiotracer. The application of the model leads to the extraction of the kinetic parameters K_1_, k_2_, k_3_ and k_4_ that describe specific molecular processes: K_1_ reflects the carrier-mediated transport of ^18^F-FDG from plasma to tissue and k_2_ its transport back from tissue to plasma, while k_3_ represents the phosphorylation rate and k_4_ the dephosphorylation rate of the tracer. Tracer influx (K_i_) is derived from the equation K_i_ = (K_1_ × k_3_)/(k_2_ + k_3_). In the present analysis, we have focused on the most clinically relevant parameters, namely K_1_, k_3_ and influx.

Besides compartment modeling, fractal analysis, a non-compartment model, was used in order to calculate the parameter of heterogeneity and complexity, expressed by a non-integer value, so-called fractal dimension (FD). The calculation of FD is performed in each individual voxel of a VOI and is based on the box-counting procedure of chaos theory. The values of FD vary from 0 to 2, showing the more deterministic or chaotic distribution of the tracer activity via time [33].

Similar to SUV,% interval changes as a response to treatment were also calculated for the quantitative (kinetic and fractal) PET parameters.

### 2.4. Statistical Analysis

Progression-free survival (PFS) was measured from the date of interim PET/CT until disease progression or death from any cause. This way of defining PFS was chosen because several covariables evaluated in the PFS analysis were determined only at the time of interim PET. Briefly, if the calculation had started at treatment, these covariables would have needed to be included as time-dependent ones, leading to marked difficulties in the interpretation of the results. Thus, in order to avoid time-dependent covariables and lead-time-bias, covariables evaluated in time-to-event-analysis are determined before or when time starts, i.e., here at interim PET. Kaplan–Meier estimates were generated, and median PFS was estimated. Median follow-up time was determined by inverse Kaplan–Meier estimation. For univariate comparison of PFS, a log-rank test was used. For comparison of PET parameters in the hierarchical setting that multiple lesions were evaluated per patient, the linear mixed model analysis was used with a random intercept for the patient. Correspondingly, a t-test was used for comparison of PET parameters with a single measurement per patient. Statistical analysis was performed using R version 4.0.2 (The R Foundation for Statistical Computing 2020) and R packages survival and prodlim. Comparisons of PET parameters with linear mixed models or with *t*-test were performed with SAS version 9.4. (SAS Institute Inc., Cary, NC, USA, 2016). The results were considered significant for *p* values less than 0.05 (*p* < 0.05). This exploratory analysis of a total of six semiquantitative (SUV_mean_, SUV_max_) and quantitative (K_1_, k_3_, influx, FD) PET parameters in different tissues was not adjusted for multiplicity of testing.

## 3. Results

The detailed characteristics of the studied patients are presented in Table 1. The results of semiquantitative and quantitative analysis of the melanoma lesions and reference organs (thyroid gland, spleen, bone marrow) as derived from baseline and interim PET/CT are presented in the following paragraphs.

### 3.1. Melanoma Lesions

A total of 98 melanoma lesions, retrieved from whole-body studies, were semiquantitatively evaluated by means of SUV calculations. These lesions were classified into five groups based on their localization. These groups were: lymph nodes (*n* = 31), osseous (*n* = 10), pulmonary (*n* = 16), mesenterial/abdominal (including hepatic) (*n* = 12), and soft tissue metastases (*n* = 29). Respectively, 55 lesions included in the field of view of the dynamic PET/CT acquisition (thorax and upper abdomen) were evaluated by means of quantitative (kinetic and fractal) analysis. These lesions were grouped as follows: lymph nodes (*n* = 15), osseous (*n* = 5), pulmonary (*n* = 13), mesenterial/abdominal (incl. hepatic) (*n* = 7), and soft tissue metastases (*n* = 15).

Two types of approaches were applied regarding the quantification of melanoma lesions:

1. The mean value of each PET parameter derived from the hottest melanoma lesions—defined as the melanoma lesions with the highest SUV_max_ in the baseline examination—was calculated per patient. Up to a maximum of five lesions in total were assessed for each patient. The results for this type of analysis are presented in Table 2
2. The value of each PET parameter derived from the single hottest (highest SUV_max_ in the baseline examination) lesion of each patient was evaluated. The results for this type of analysis are presented in Table 3.

Briefly, with regard to SUV calculations, melanoma lesions showed an up to 30% decrease of the median values of SUV_mean_ and SUV_max_ as a response to treatment according to both approaches. Similarly, ^18^F-FDG kinetic parameters responded with a decrease of their median values. Only minor changes were observed for the parameter FD. In total, no statistically significant changes took place in melanoma lesions as a response to treatment.

Considering the differences between the treatment regimens—in particular, between monotherapy and combination therapy—regarding their mechanism of action, efficacy, response patterns and toxicity profile, we proceeded to dichotomization of the patient cohort according to the therapy applied. Separate analyses were performed for the two groups of patients (monotherapy vs. combination therapy), including all parameters. The calculations of the semiquantitative and quantitative parameters of the melanoma lesions derived from baseline and follow-up PET/CT revealed no significant differences between the two groups. For the sake of space, the respective descriptive statistics are provided in the Supplementary Materials (Supplementary Tables S1 and S2).

Further, the metastatic lesions were classified in the above-mentioned five groups based on their localization (lymphatic, osseous, pulmonary, mesenterial/abdominal, soft tissue). Statistical analysis revealed no significant differences between the different sites for almost all parameters and both time-points of PET/CT scanning (baseline, interim). The only exception was the parameter FD, for which some variations in the mean values were noted. However, this finding should not be overinterpreted since the comparisons were not adjusted for multiplicity. The results of this analysis are presented in the Supplementary Materials (Supplementary Table S3).

**Figure 1:**
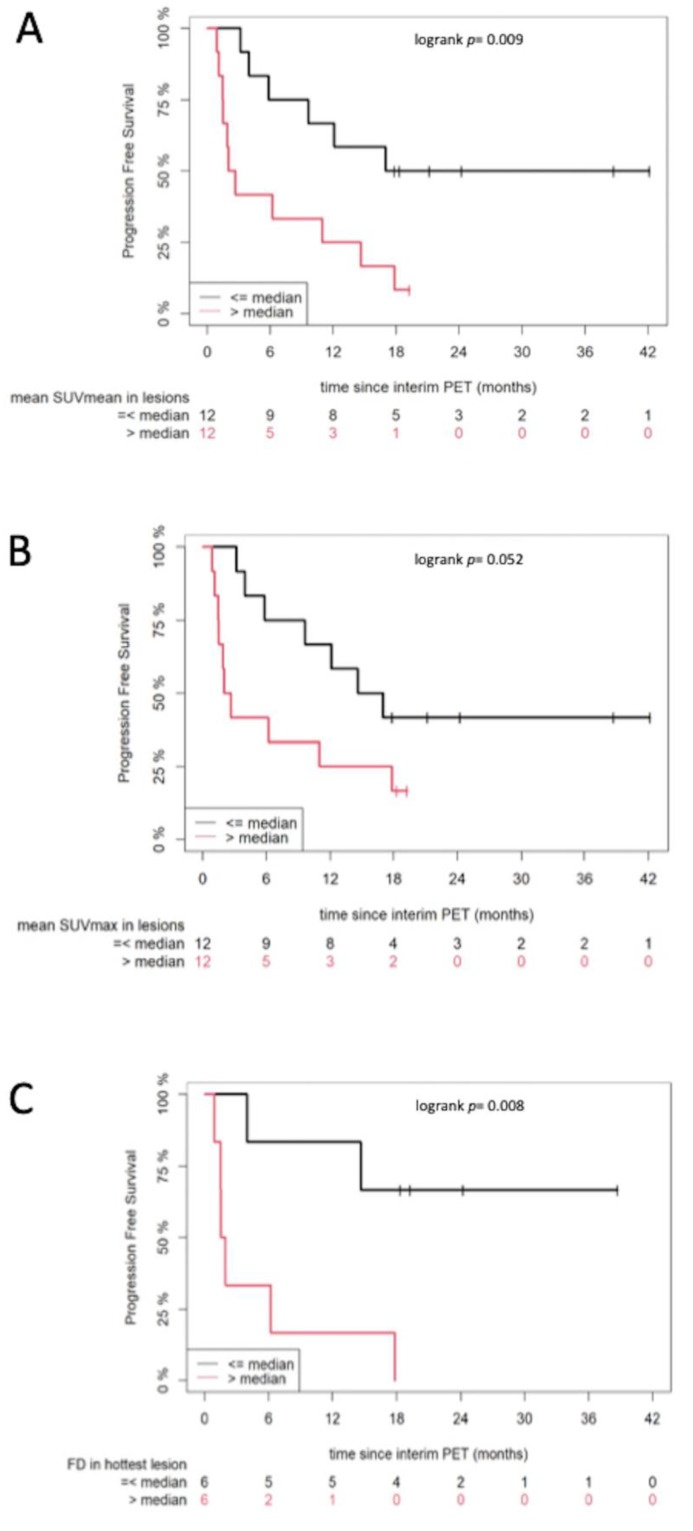
Kaplan–Meier estimates of progression-free survival (PFS) according to different parameters derived from interim PET/CT. These parameters are SUV_mean_ (**A**) and SUV_max_ (**B**), derived from the mean value of the hottest melanoma lesions for each patient (up to five lesions), as well as according to FD (**C**), derived from the single hottest melanoma lesion for each patient. The numbers of patients at risk in each group and for the respective time-points are shown below the plots.

Median follow-up [95% CI] of the patient cohort from interim PET/CT was 24.2 months [19.3–41.7 months]. All semiquantitative and quantitative PET parameters were dichotomized at the median to investigate their effect on PFS. The results of survival analysis revealed that the following parameters on interim PET/CT adversely affected PFS: SUV_mean_ (*p* = 0.009) and SUV_max_ (*p* = 0.052) derived from the mean value of the five hottest melanoma lesions for each patient (approach 1), as well as FD of the hottest lesion in each patient (*p* = 0.008) (approach 2) (Figure 1). In particular, patients with parameters above the median values showed shorter survival than those with parameters below the median. No parameter from baseline PET/CT had a significant effect on survival.

### 3.2. Reference Organs

In analogy to melanoma lesions, assessment of the semiquantitative and quantitative parameters was also performed for the reference organs. In particular, regarding SUV, the thyroid gland showed an increase of the median values of SUV_mean_ (+10%) and SUV_max_ (+15%) as a response to treatment. The respective interval SUV changes in the bone marrow, and the spleen were very small. The results of quantitative analysis for reference organs are presented in Table 4, Table 5 and Table 6. No statistically significant changes took place in any organ as a response to treatment.

Similar to melanoma lesions, the patient cohort was divided into two groups according to the treatment applied (monotherapy vs. combination therapy), and separate analyses were also performed regarding the reference organs. The calculations of the semiquantitative and quantitative parameters derived from baseline and follow-up PET/CT revealed no significant differences between the groups of treatment. The respective descriptive statistics are provided in the Supplementary Materials (Supplementary Tables S1 and S2).

**Figure 2:**
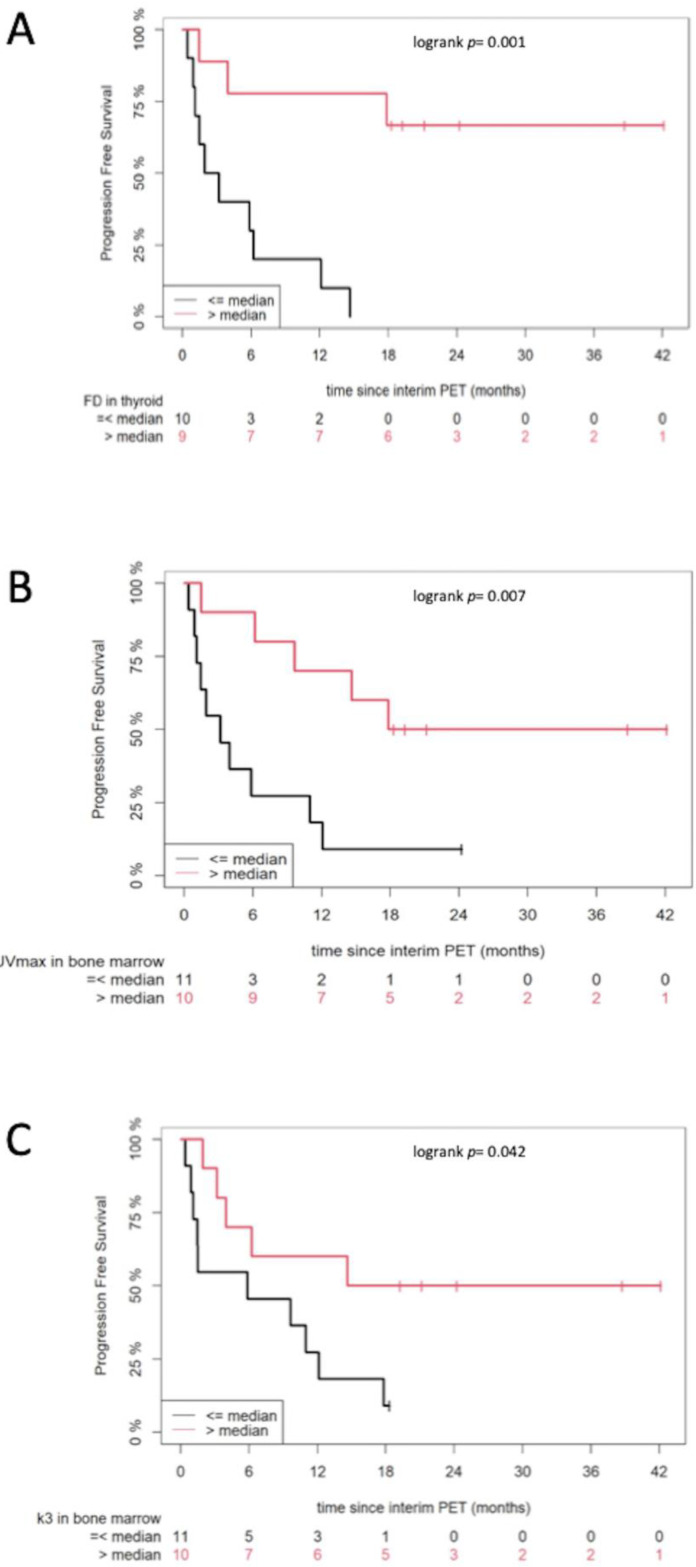
Kaplan–Meier estimates of PFS according to FD derived from the thyroid (**A**), as well as SUV_max_ (**B**) and k_3_ (**C**) derived from the bone marrow, on interim positron emission tomography/computed tomography (PET/CT). The numbers of patients at risk in each group and for the respective time-points are shown below the plots.

Survival analysis revealed that the following parameters derived from interim PET/CT dichotomized at the median positively affected PFS: FD of the thyroid, SUV_max_ of the bone marrow, and k_3_ of the bone marrow (Figure 2). In particular, patients with parameters above the respective median values showed longer survival than those with parameters below the median. Similar to tumor lesions, none of the parameters derived from baseline PET/CT were prognostic for survival.

## 4. Discussion

The vast majority of oncological treatment response evaluations are based on changes in tumor size [34,35], which represent, however, the last step in a series of metabolic and functional processes and are, thus, considered late-occurring events. Moreover, due to their unique mechanism of action, ICIs can generate novel, previously unreported response patterns, whose reliable interpretation poses challenges in conventional imaging modalities. In this framework, the role of ^18^F-FDG PET/CT is increasingly investigated since it can detect therapy effects on a metabolic level before morphological changes take place [36]. Tumor imaging by means of ^18^F-FDG PET/CT is based on the metabolic programming of cancer cells, in particular on their preference to metabolize glucose by aerobic glycolysis, rather than oxidative phosphorylation, for ATP generation so-called Warburg effect [37]. Apart from its well-documented affinity for tumor tissue, ^18^F-FDG also accumulates at sites of infiltration by cytotoxic T-cells, the main immune cells involved in the antitumor mechanism of ICIs, allowing their visualization by means of PET/CT [23,38].

These features render imaging with ^18^F-FDG PET/CT attractive for immunotherapy monitoring, but on the other hand, they may partly lead to interpretation pitfalls, including false-positive findings, such as the phenomenon of pseudoprogression [9,39] or the emergence of irEs at non-tumor tissue. One strategy to tackle this limitation is the application of more specific imaging biomarkers than ^18^F-FDG, currently under investigation [40,41,42,43]. In the absence, however, of such tracers widely available for clinical use, objective quantification of tracer uptake is gaining significance.

SUV is a widely used semiquantitative PET metric because of its simplicity and reproducibility [44]. These attributes have led to its broad investigation as a potential biomarker of immunotherapy response assessment [19,20,22,45,46]. Another quantification approach involves the employment of dynamic PET/CT protocols, which offer the possibility of more detailed calculations of tracer kinetics compared to standard imaging. In the case of ^18^F-FDG, the performance of early, dynamic PET/CT studies and the subsequent application of compartment and fractal analysis allow the investigation of dedicated parameters of the tracer’s metabolism [47]. However, data on dynamic, quantitative ^18^F-FDG PET/CT in ICIs’ monitoring remain scarce [25]. This is mainly attributed to the difficulty of implementation of dynamic PET/CT in routine clinical practice: first, it is more time-consuming than conventional, static PET/CT scanning since the acquisition of a full dynamic PET scan is a 60-min process. Moreover, sophisticated software tools are required for the performance of the complicated compartment and fractal analysis in order to evaluate the dynamic data. Despite these obstacles, in the present study, both approaches, the semiquantitative and the quantitative, were utilized in an attempt to investigate the predictive role of “full” quantitative ^18^F-FDG PET/CT analysis on the survival of metastatic melanoma patients treated with PD-1 blockade.

Semiquantitative analysis of ^18^F-FDG uptake revealed that median SUV values of melanoma lesions decreased by 4% to 30% as a response to anti-PD-1 treatment. Moreover, SUV values derived from interim PET/CT adversely affected PFS. These findings are in line with the results by Ito et al. in 60 melanoma patients treated with ipilimumab and examined with PET/CT before and after treatment. The authors of that study observed a significant association between changes in tumor SUV and overall survival (OS) [22]. Moreover, Nobashi et al. showed in a heterogeneous cohort of 40 patients undergoing immunotherapy that clinical responders demonstrated a significant decrease of SUV_max_ between baseline and restaging PET/CT [47]. On the other hand, our group had previously failed to show a significant correlation between SUV changes and clinical benefit to ipilimumab [25,45]; instead, the number of a newly emerging, ^18^F-FDG-avid lesions could more reliably serve patient classification to progressive disease and, thus, predict response to immunotherapy (sensitivity = 84%, specificity = 100%). Based on this, a new set of response criteria was suggested, the PET response evaluation criteria for immunotherapy (PERCIMT) [45]. The PERCIMT-based dichotomization of the herein studied cohort into metabolic responders and nonresponders showed that the first ones had a significantly longer PFS than the latter [26]. Interestingly, Cho et al. found in a group of 20 melanoma patients treated with different ICIs that an increase in ^18^F-FDG tumor uptake in PET/CT performed at 3–4 weeks into therapy may correlate with clinical benefit, reaching a 100% sensitivity, a 93% specificity, and a 95% accuracy. This observation led to the introduction of the PET/CT Criteria for early prediction of Response to Immune checkpoint inhibitor Therapy (PECRIT), which combines anatomic and functional imaging data collected at follow-up PET/CT [20]. Based on these mixed—and partly contradictory—results, we can assume that SUV changes in tumor lesions may have a predictive value in immunotherapy outcome, but SUV is far from being considered a perfect prognostic indicator. SUV changes may be helpful in assessing partial or complete response but do not enable a reliable distinction between borderline cases of stable and progressive disease. Furthermore, several issues still remain to be clarified, mostly deriving from the nonspecific nature of ^18^F-FDG, which, as mentioned above, can accumulate in both tumor lesions and ICIs’-induced sites of inflammation.

Quantitative analysis of the dynamic PET/CT data of melanoma lesions after application of compartment modeling revealed a decreasing, not statistically significant, pattern of all ^18^F-FDG kinetic parameters regarding their interval changes as a response to therapy. Nevertheless, survival analysis did not reveal any effect of these parameters on PFS. This finding is in accordance with previously published results by our group on 25 metastatic melanoma patients undergoing ipilimumab monotherapy. In that study, due to the lack of survival data at the time point of evaluation, the patients’ best clinical response—based on a combination of clinical, biochemical and imaging data—was used as a reference. Similar to the present study, no significant differences between responders and nonresponders to ipilimumab were observed [25].

On the other hand, a quantitative tumor parameter found to negatively influence survival was the degree of ^18^F-FDG heterogeneity, reflected by the index FD. FD is calculated with the box-counting method and provides an estimate of the complexity of a dimensional structure [33]. In the present analysis, higher FD values and subsequently a more heterogeneous distribution of the glucose analog in melanoma lesions early during anti-PD-1 treatment had an adverse effect on PFS. The potential of fractal mathematics in oncological research and practice remains relatively unknown to most clinicians. Nevertheless, the hitherto rather limited but steadily growing literature in the field implies a possible contribution of the investigation of tumor heterogeneity, utilizing fractal principles on PET images, in tumor characterization and patient prognosis [48,49,50,51].

Aside from melanoma lesions, we also assessed the thyroid gland, whose dysfunction is a frequent endocrine irAE induced by PD-1 inhibitors [52,53], as well as primary and secondary lymphoid organs, given the critical impact of systemic immune responses for effective cancer immunotherapy [54].

The quantitative analysis of the thyroid showed a non-negligible SUV increase in the gland as a response to treatment, which is in line with previously published results [46]. Interestingly, patients with higher FD on interim PET/CT showed significantly longer PFS, suggesting a positive survival effect of the heterogeneity of ^18^F-FDG distribution in the thyroid during PD-1 blockade. Although the pathophysiological basis for these findings cannot be easily clarified, we note the documented association between the development of thyroid dysfunction and an improvement in survival of patients treated by PD-1 blockade [55]. Importantly, in a recently published meta-analysis, a significant association between the development of endocrine irAEs, among which thyroid dysfunction, and a favorable benefit on survival was highlighted [56].

The bone marrow represents the main hematopoietic organ in adults and a supportive organ to immune cell function [57]. In this context, the assessment of bone marrow glucose metabolism during cancer immunotherapy is of interest. Nobashi et al. reported on a decrease of bone marrow SUV_max_ after immunotherapy in patients with different tumors, in both clinical responders and nonresponders to treatment [46]. On the other hand, Schwenk et al. found a significant increase of glucose metabolism in the bone marrow of responders, while a decreased ^18^F-FDG uptake was observed in nonresponders [58]. In our analysis, the parameters SUV_max_ and k_3_ derived from interim PET/CT positively affected the patient suggesting that the degree of activation of the bone marrow during PD-1 blockade, as reflected by respective changes in ^18^F-FDG uptake and kinetics, may play a predictive role in treatment outcome of metastatic melanoma. Of course, this needs to be validated by further studies, including larger cohorts.

Finally, we evaluated the glucose metabolism in the spleen, the largest secondary lymphoid organ and blood-filtering unit in the body [59,60,61]. The spleen is involved in response to systemic inflammatory stimuli, which, in terms of PET imaging, can be observable by increased ^18^F-FDG uptake in the organ [58,62,63,64]. In the present analysis, only minor SUV changes were observed as a response to treatment. Moreover, no parameter both on the baseline and interim PET/CT had any effect on patient survival. These findings are supportive of previously published results of our group and others, suggesting a rather poor contribution of spleen metabolism, studied by ^18^F-FDG PET/CT, in monitoring and prediction of outcome in melanoma patients under immunotherapy [46,58,65].

Taken together, there are two major findings from our study. First, the SUV values, as well as FD of metastatic melanoma lesions, seem to have an adverse effect on PFS already after the application of two cycles of PD-1 blockade. Second, we show for the first time that quantitative parameters derived from specific reference tissues, namely the thyroid and the bone marrow, can also be predictive of PFS, but in this case with a positive effect. Although these findings could suggest the wider usage of dynamic PET/CT in oncological clinical practice, we are aware of the practical considerations that accompany this approach, rendering its routine application burdensome. In view of this, we can recommend for the time being the use of dynamic PET/CT—performed in combination with conventional, static, whole-body PET/CT—for selected oncological cases, most likely in terms of investigating treatment response to specific therapeutic regimens. By complementing the information offered by conventional imaging with the multiparametric, pharmacokinetic data extracted by dynamic PET/CT, the diagnostic certainty of the reading physician could be enhanced, while our understanding of the pathophysiology involved in the natural history of the tumor and its response to treatment would be improved. Moreover, the future perspective of quantitative, dynamic PET/CT seems prosperous: the recent advent of new PET/CT scanners, which allow dynamic studies over several bed positions by using a continuous bed movement, as well as the introduction of new PET/CT scanners with an extended field of view (>1 m) will facilitate the use of dynamic PET protocols and reduce the whole acquisition time, making dynamic PET/CT an attractive and cost-effective approach in oncological imaging [66].

Our study has some limitations. Foremost, the relatively small number of studied patients does not allow the drawing of more firm conclusions. This is, however, mainly attributed to the strict inclusion criteria applied in the study. The lack of histological validation of the vast majority of the PET/CT positive findings constitutes another limitation of our analysis. However, this is usually not possible in the clinical setting. Moreover, despite the fact that a two-bed position protocol, which allows the study of a relatively large field of view of 44 cm, was used, the dynamic PET/CT acquisition was confined only in the anatomic area of the thorax/upper abdomen, not allowing for whole-body dynamic studies to be performed.

## 5. Conclusions

We explored the prognostic significance of quantitative, dynamic ^18^F-FDG PET/CT performed early during PD-1 blockade in metastatic melanoma. In this context, we assessed the impact of semiquantitatively and quantitatively derived parameters on the PFS of 25 melanoma patients. Our results showed that several parameters derived from interim PET/CT, performed as early as after administration of two cycles of ICIs, had a significant impact on survival. In particular, the intensity of ^18^F-FDG uptake (SUV_mean_, SUV_max_) and the heterogeneity of tracer distribution (FD) in melanoma lesions adversely affected PFS. On the other hand, the degree of ^18^F-FDG heterogeneity (FD) in the thyroid, as well as the intensity ^18^F-FDG uptake (SUV_max_) and phosphorylation rate (k_3_) in the bone marrow, were associated with a positive effect on PFS. These findings highlight the potential predictive role of quantitative, dynamic, interim PET/CT—performed in combination with conventional, static, whole-body PET/CT—in metastatic melanoma patients under PD-1 blockade. Dynamic PET/CT could be performed in selected oncological cases in combination with static, whole-body PET/CT in order to enhance the diagnostic certainty offered by conventional imaging and yield additional information regarding specific molecular and pathophysiological mechanisms involved in tumor biology and response to treatment.

## Figures and Tables

**Table 1:** Patient characteristics.

Patient Number	Age	Gender	LDH at Baseline (U/I)	Treatment	Distribution of Metastases	Progression	PFS (Months)
1	56	F	770	Ipilimumab/nivolumab	Pulmonary, hepatic, osseous, lymphatic, mesenteric, pancreas	Yes	1.5
2	34	F	247	Ipilimumab/nivolumab	Cutaneous/subcutaneous, hepatic, pulmonary, lymphatic, adrenal, osseous, soft tissue	No	17.9
3	46	F	166	Ipilimumab/nivolumab	Pulmonary, soft tissue	Yes	4.0
4	54	M	218	Ipilimumab/nivolumab	Osseous, lymphatic	Yes	3.2
5	53	M	275	Ipilimumab/nivolumab	None	Yes	0.4
6	50	F	344	Ipilimumab/nivolumab	Mesenteric	No	19.3
7	59	M	204	Ipilimumab/nivolumab	Pulmonary	No	38.7
8	44	F	340	Ipilimumab/nivolumab	Soft tissue, lymphatic	No	18.3
9	60	F	269	Ipilimumab/nivolumab	Pulmonary, lymphatic	No	21.2
10	48	F	246	Ipilimumab/nivolumab	Pulmonary, osseous	No	24.2
11	55	F	224	Nivolumab	Mesenteric, soft tissue	Yes	6.2
12	84	F	195	Pembrolizumab	Soft tissue, lymphatic	Yes	11.0
13	79	M	205	Pembrolizumab	Pulmonary, lymphatic, subcutaneous	Yes	2.0
14	20	F	186	Ipilimumab/nivolumab	Soft tissue, lymphatic	Yes	17.8
15	52	M	275	Pembrolizumab	Hepatic, lymphatic, subcutaneous, soft tissue	No	42.1
16	52	M	170	Pembrolizumab	Lymphatic	Yes	14.6
17	53	F	260	Nivolumab	Lymphatic, subcutaneous	Yes	9.6
18	65	M	201	Pembrolizumab	Pulmonary, lymphatic	Yes	1.9
19	67	F	290	Pembrolizumab	Lymphatic, mesenteric, pulmonary	Yes	5.9
20	55	M	200	Pembrolizumab	Soft tissue, lymphatic, cutaneous/subcutaneous	Yes	1.5
21	58	M	183	Ipilimumab/nivolumab	Pulmonary, lymphatic, cutaneous/subcutaneous	Yes	17.0
22	50	M	195	Ipilimumab/nivolumab	Lymphatic, osseous	Yes	12.1
23	80	M	364	Pembrolizumab	Osseous, pulmonary, adrenal	Yes	0.9
24	47	F	256	Nivolumab	Subcutaneous, lymphatic, soft tissue, osseous, pulmonary	Yes	1.1
25	47	M	271	Nivolumab	Lymphatic, mesenteric, osseous, soft tissue	Yes	2.7

F, female; M, male; LDH, lactate dehydrogenase; PFS, progression-free survival.

**Table 2:** Median values of the ^18^F-FDG semiquantitative and quantitative parameters before and after two cycles of anti-programmed cell death protein 1 (PD-1) therapy as well as their interval changes (%) as a response to treatment. The values are derived from the mean of the hottest melanoma lesions, up to a maximum of five lesions total for each patient. The units of parameters K_1_, k_3_ and influx are 1/min. SUV_mean_, SUV_max_ and FD have no unit.

Parameter	Baseline PET/CT	Interim PET/CT	Interval Change (%)
SUV_mean_	6.9	5.7	−17%
SUV_max_	9.8	9.4	−4%
K_1_	0.19	0.15	−15%
k_3_	0.19	0.18	−5%
Influx (K_i_)	0.05	0.03	−40%
FD	1.19	1.21	+2%

**Table 3:** Median values of the ^18^F-FDG semiquantitative and quantitative parameters, derived from the hottest melanoma lesion for each patient, before and after two cycles of anti-PD-1 therapy, as well as their interval changes (%) as a response to treatment. The units of parameters K_1_, k_3_ and influx are 1/min. SUV_mean_, SUV_max_ and FD have no unit.

Parameter	Baseline PET/CT	Interim PET/CT	Interval Change (%)
SUV_mean_	11.0	7.7	−30%
SUV_max_	15.1	12.7	−16%
K_1_	0.27	0.14	−48%
k_3_	0.24	0.22	−8%
Influx (K_i_)	0.08	0.06	−25%
FD	1.29	1.28	−1%

**Table 4:** Median values of the ^18^F-FDG semiquantitative and quantitative parameters, derived from the thyroid, before and after two cycles of anti-PD-1 therapy as well as their interval changes (%) as a response to treatment. The units of parameters K_1_, k_3_ and influx are 1/min. SUV_mean_, SUV_max_ and FD have no unit.

Parameter	Baseline PET/CT	Interim PET/CT	Interval Change (%)
SUV_mean_	2.1	2.3	+10%
SUV_max_	2.6	3.0	+15%
K_1_	0.32	0.28	−13%
k_3_	0.08	0.22	+64%
Influx (K_i_)	0.02	0.02	0
FD	1.06	1.09	+3%

**Table 5:** Median values of the ^18^F-FDG semiquantitative and quantitative parameters, derived from the bone marrow, before and after two cycles of anti-PD-1 therapy as well as their interval changes (%) as a response to treatment. The units of parameters K_1_, k_3_ and influx are 1/min. SUV_mean_, SUV_max_ and FD have no unit.

Parameter	Baseline PET/CT	Interim PET/CT	Interval Change (%)
SUV_mean_	2.3	2.4	+4%
SUV_max_	3.1	3.2	+3%
K_1_	0.18	0.13	−28%
k_3_	0.09	0.12	+33%
Influx (K_i_)	0.03	0.02	−33%
FD	1.06	1.07	+1%

**Table 6:** Median values of the ^18^F-FDG semiquantitative and quantitative parameters, derived from the spleen, before and after two cycles of anti-PD-1 therapy, as well as their interval changes (%) as a response to treatment. The units of parameters K_1_, k_3_ and influx are 1/min. SUV_mean_, SUV_max_ and FD have no unit.

Parameter	Baseline PET/CT	Interim PET/CT	Interval Change (%)
SUV_mean_	2.3	2.2	−4%
SUV_max_	3.2	3.2	0
K_1_	0.40	0.25	−38%
k_3_	0.07	0.05	−29%
Influx (K_i_)	0.03	0.03	0
FD	1.10	1.09	−1%

## Data Availability

The authors confirm that the data supporting the findings of this study are available within the article and its Supplementary Materials.

## Supplementary Materials

The following are available online at https://www.mdpi.com/2072-6694/13/5/1019/s1, Table S1: Median values of the ^18^F-FDG semi-quantitative and quantitative parameters before and after two cycles of anti-PD-1 monotherapy. Table S2: Median values of the ^18^F-FDG semi-quantitative and quantitative parameters before and after two cycles of combination therapy. Table S3: Median values of the ^18^F-FDG semi-quantitative and quantitative parameters before and after two cycles of anti-PD-1 therapy, derived from melanoma lesions classified according to their localisation.
