# Supplementary material for: Quantitative Dynamic 18F-FDG PET/CT in Survival Prediction of Metastatic Melanoma under PD-1 Inhibitors
Source: Cancers (Basel). 2021 Mar 1;13(5):1019. doi: 10.3390/cancers13051019 (PMC7957728; doi:10.3390/cancers13051019)
Supplement: Supplementary file 1 [file cancers-13-01019-s001.pdf]

Supplementary Table S1. Median values of the ^{18}F-FDG semi-quantitative and quantitative parameters before and after two cycles of anti-PD-1 monotherapy (nivolumab or pembrolizumab) (n=12 patients). The values are derived from the mean of the hottest melanoma lesions per-patient as well as reference tissues. The units of parameters K_1, k_3 and influx are 1/min. SUV_{mean}, SUV_{max} and FD have no unit.

	SUV_{mean}	SUV_{max}	K_1	k_3	Influx (K_i)	FD
Baseline PET/CT						
Melanoma lesions	6.4	9.8	0.22	0.19	0.03	1.20
Thyroid	1.9	2.4	0.25	0.08	0.02	1.05
Bone marrow	2.6	3.4	0.15	0.08	0.02	1.08
Spleen	2.3	3.3	0.33	0.05	0.03	1.10
Interim PET/CT						
Melanoma lesions	7.4	11.6	0.12	0.18	0.03	1.21
Thyroid	1.8	2.4	0.17	0.06	0.01	1.05
Bone marrow	2.6	3.2	0.13	0.11	0.02	1.08
Spleen	2.1	3.1	0.24	0.05	0.02	1.09

Supplementary Table S2. Median values of the ^{18}F-FDG semi-quantitative and quantitative parameters before and after two cycles of combination therapy of ipilimumab/nivolumab (n=13 patients). The values are derived from the mean of the hottest melanoma lesions per-patient as well as reference tissues. The units of parameters K_1, k_3 and influx are 1/min. SUV_{mean}, SUV_{max} and FD have no unit.

	SUV_{mean}	SUV_{max}	K_1	k_3	Influx (K_i)	FD
Baseline PET/CT						
Melanoma lesions	8.3	10.6	0.17	0.20	0.05	1.18
Thyroid	2.2	2.9	0.50	0.08	0.06	1.08
Bone marrow	2.2	2.8	0.47	0.09	0.08	1.05
Spleen	2.1	2.9	0.56	0.07	0.09	1.09
Interim PET/CT						
Melanoma lesions	4.7	6.1	0.15	0.18	0.04	1.19
Thyroid	2.5	3.8	0.33	0.06	0.02	1.10
Bone marrow	2.4	3.2	0.14	0.13	0.03	1.05
Spleen	2.3	2.2	0.36	0.05	0.03	1.11

Supplementary Table S3. Median values of the ^{18}F-FDG semi-quantitative and quantitative parameters before and after two cycles of anti-PD-1 therapy, derived from melanoma lesions classified according to their localisation. The units of parameters K_1, k_3 and influx are 1/min. SUV_{mean}, SUV_{max} and FD have no unit.

	SUV_{mean}	SUV_{max}	K_1	k_3	Influx (K_i)	FD
Baseline PET/CT						
Lymph nodes	8.6	12.0	0.17	0.16	0.03	1.18
Osseous	10.7	11.1	0.22	0.22	0.06	1.28
Pulmonary	5.5	7.2	0.19	0.08	0.03	1.10
Abdominal	9.0	13.1	0.28	0.09	0.04	1.26
Soft tissue	8.0	8.5	0.13	0.29	0.06	1.21
Interim PET/CT						
Lymph nodes	5.7	8.0	0.13	0.12	0.03	1.13
Osseous	7.3	8.0	0.10	0.11	0.04	1.25
Pulmonary	5.7	9.5	0.16	0.19	0.03	1.16
Abdominal	8.4	11.0	0.16	0.13	0.04	1.24
Soft tissue	8.0	11.8	0.22	0.13	0.06	1.28
